# Association of Single Nucleotide Polymorphisms on Locus 18q21.1 in the Etiology of Nonsyndromic Cleft Lip Palate (NSCLP) in Indian Multiplex Families

**DOI:** 10.1055/s-0041-1723087

**Published:** 2021-02-19

**Authors:** Praveen Kumar Neela, Gosla Srinivas Reddy, Akhter Husain, Vasavi Mohan, Sravya Thumoju, Rajeshwari BV

**Affiliations:** 1 Department of Orthodontics, Kamineni Institute of Dental Sciences, Narketpally, Telangana, India and GSR Institute of Craniomaxillofacial and Facial Plastic Surgery, Hyderabad, Telangana, India; 2 Department of Craniofacial Surgery, GSR Institute of Craniofacial Surgery, Hyderabad, India; 3 Department of Orthodontics, Yenepoya Dental College, Yenepoya University, Mangaluru, Karnataka, India; 4 Department of Genetics and Molecular Medicine, Vasavi Medical and Research Centre, Hyderabad, Telangana, India; 5 Department of Obstetrics & Gynaecology, Surabhi Institute of Medical Sciences, Telangana, India

**Keywords:** cleft lip palate, chromosome, SNP

## Abstract

**Background** Cleft lip palate (CLP) is a common congenital anomaly with multifactorial etiology. Many polymorphisms at different loci on multiple chromosomes were reported to be involved in its etiology. Genetic research on a single multigenerational American family reported 18q21.1 locus as a high-risk locus for nonsyndromic CLP (NSCLP). However, its association in multiple multiplex families and Indian population is not analyzed for its association in NSCLP.

**Aim** This study was aimed to evaluate whether high-risk single nucleotide polymorphisms (SNPs) on chromosome 18q21.1 are involved in the etiology of NSCLP in multiplex Indian families.

**Materials and Methods** Twenty multigenerational families affected by NSCLP were selected for the study after following inclusion and exclusion criteria. Genomic DNA was isolated from the affected and unaffected members of these 20 multiplex families and sent for genetic analysis. High-risk polymorphisms, such as rs6507872 and rs8091995 of *CTIF* , rs17715416, rs17713847 and rs183559995 of *MYO5B* , rs78950893 of *SMAD7* , rs1450425 of *LOXHD1* , and rs6507992 of *SKA1* candidate genes on the 18q21.1 locus, were analyzed. SNP genotyping was done using the MassARRAY method. Statistical analysis of the genomic data was done by PLINK.

**Results** Polymorphisms followed the Hardy–Weinberg equilibrium. In the allelic association, all the polymorphisms had a *p* -value more than 0.05. The odds ratio was not more than 1.6 for all the SNPs.

**Conclusion** High-risk polymorphisms, such as rs6507872 and rs8091995 of *CTIF* , rs17715416, rs17713847 and rs183559995 of *MYO5B* , rs78950893 of *SMAD7* , rs1450425 of *LOXHD1* , and rs6507992 of *SKA1* in the locus 18q21.1, are not associated with NSCLP in Indian multiplex families.

## Introduction

One of the important congenital defects seen in the orofacial region is cleft lip palate (CLP). An infant is born with a cleft lip or palate somewhere on the planet in every 2 minutes according to a study by World Health Organization (WHO). 1 The world wide surveys show that the frequency of cleft lip and palate varies significantly from one country to another. It is lowest in Africans (1:2,500) while the North American Indians and East Asians have the highest prevalence (1:500). In an Indian study by Reddy et al, 2 the incidence of clefts reported was 1:800 to 1:1,000 and three infants are born with some type of cleft every hour. CLP can be syndromic or nonsyndromic (NSCLP). Approximately 70% of the cleft lip and palate cases are nonsyndromic and occur as isolated cases, whereas the remaining 30% of clefts are syndromic and are associated with some other anomalies. 3 Our knowledge of the etiology and pathogenesis of nonsyndromic variants yet remain relatively deficient. Etiology of CLP is multifactorial which includes genetic causes, malnutrition, endocrine disorders, infection, trauma, and consanguinity. Roughly, 20% of the CLP showed consanguinity of their parents, while the percentage of familial cases is 3.5% of all the cleft cases. 4 About 600 syndromes are characterized by some form of cleft phenotype. 5

The research on the genetics of CLP used both association analysis and linkage analysis to determine the genetic determinants of oral and facial clefts. The results of candidate gene-based association studies, performed in diverse populations, have been mostly inconclusive or conflicting, with only a few candidate loci implicated in cleft phenotypes. These studies discovered multiple candidate genes linked to *NSCLP* such as *IRF6* , *MSX1* , *CRISPLD2* , *ABC4* , *RARA* , *TGFα* , *TGFβ* , *p63* , *MYH9* , *BCL3* , *MTHFR* , *TGFB2* , *SATB2* , *P63* , *MSX2* , *FOXE1* , *BMP4* , *PAX7* , *PVRL1* , *TGFB3* , *RARA* , *RUNX2* , *BCL3* , *TGFB1* , *TBX1* , and *BCL3* . 6 7 8 9 10

Genome-wide association studies (GWAS) done on families, isolated cleft patients, revealed significant evidence of linkage at different loci on multiple chromosomes. Some of them include 7p21.3, 14q32.32, 9p23, 3q26.33, 10q25.1, 18q21.1, 6p12.3, and 4q28.1. Beiraghi et al 11 in 2006, after a GWAS on single, multigenerational family, reported evidence of linkage at 18q21.1 locus. Later, in a fine-mapping study on the same family, Mitra et al 12 in 2016 reported high-risk variants on the same locus associated with NSCLP. However, no study has evaluated this high-risk region in multiple multiplex or multigenerational families and our Indian population. Therefore, the present study conducted to know the association of high-risk markers, such as rs6507872 and rs8091995 of *CTIF* , rs17715416, rs17713847 and rs183559995 of *MYO5B* , rs78950893 of *SMAD7* , rs1450425 of *LOXHD1* , and rs6507992 of *SKA1* gene present on locus 18q21.1, in the etiology of NSCLP in Indian multiplex families. The study hypothesized that SNPs selected from this high-risk region for NSCLP are not involved in the etiology of NSCLP in multiplex families our population.

## Materials and Methods

**Fig. 1:**
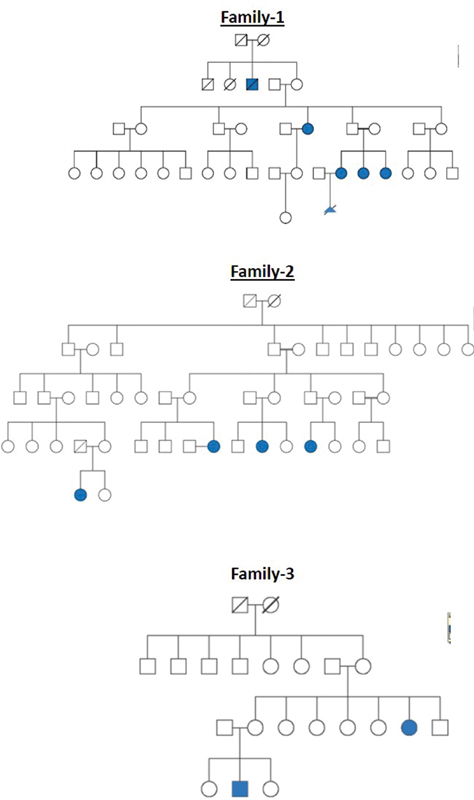
Pedigree chart for families 1, 2, and 3.

The Institutional Review Board of GSR Institute of Craniofacial Surgery approved the research. The research procedures were performed following the principles of the Declaration of Helsinki. Multiplex families with cleft lip with or without palate were selected after going through the medical records of the institute. Patients with only nonsyndromic cleft lip or without palate were included. Syndromic CLP cases, chromosomal aberrations, associated malformations, and mental retardation were excluded. Twenty multigenerational families, according to the above criteria, were selected based on power calculation for family-based association studies. 13 All these cleft families were selected from a high-volume cleft center where patients from different parts of the country come for treatment. The pedigree charts for all the 20 families are shown in Figs. 1 2 3 4 5 6 . The number of affected patients include 1 family with five affected, 2 families with four affected, 5 families with three affected, and 12 families with two affected. A total of 50 affected and 38 unaffected participated in the research. Informed consent was taken from all the members who participated in the study. In the case of minors, either of the parent's consent was taken. In all the 20 families, unaffected patients who voluntarily participated in the research were taken as controls.

**Fig. 2:**
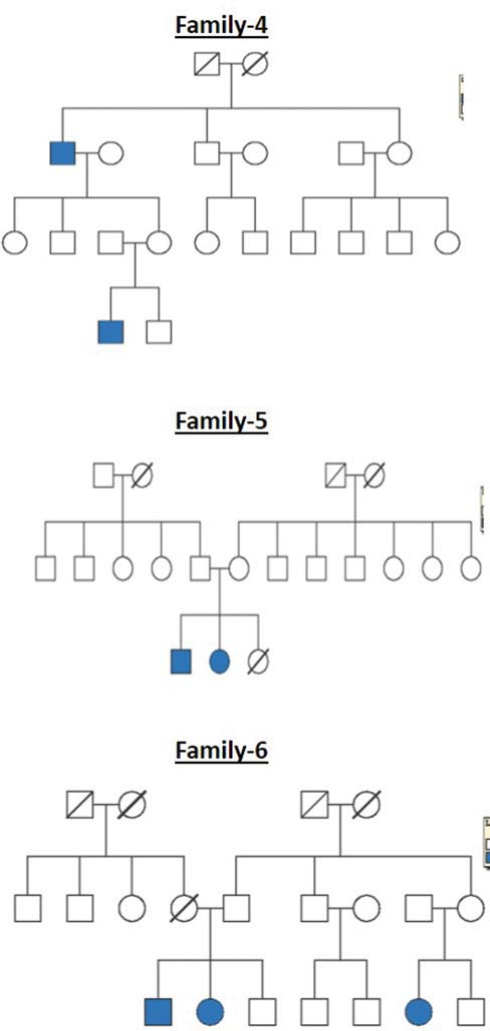
Pedigree chart for families 4, 5, and 6.

**Fig. 3:**
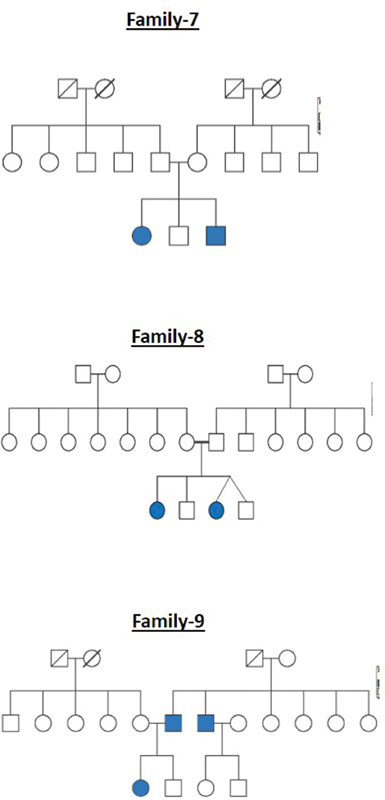
Pedigree chart for families 7, 8, and 9.

**Fig. 4:**
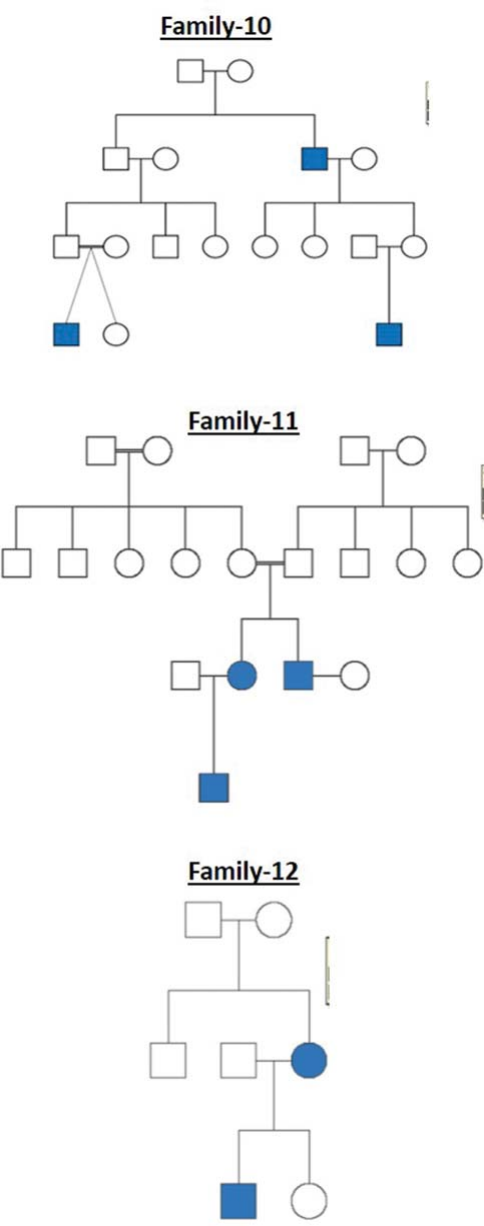
Pedigree chart for families 10, 11, and 12.

**Fig. 5:**
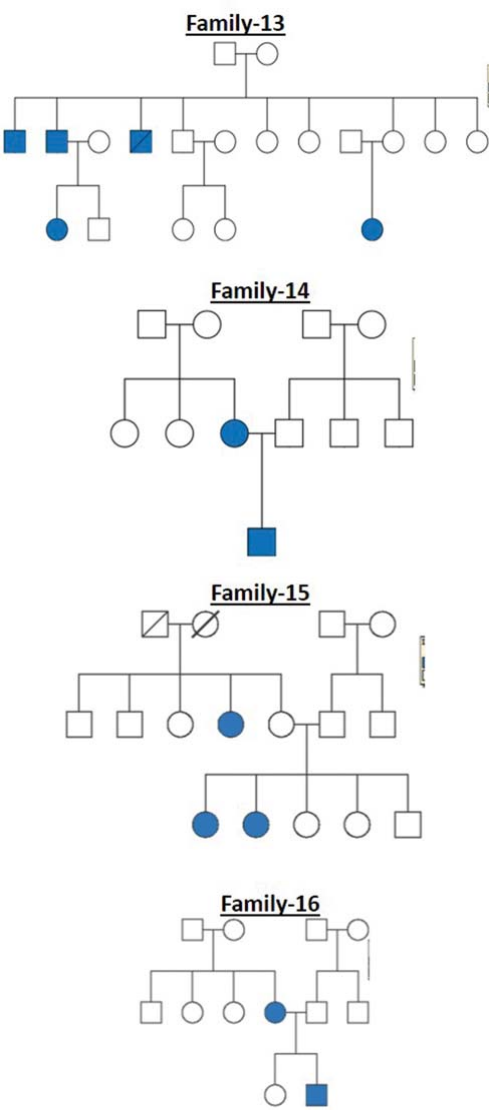
Pedigree chart for families 13, 14, 15, and 16.

**Fig. 6:**
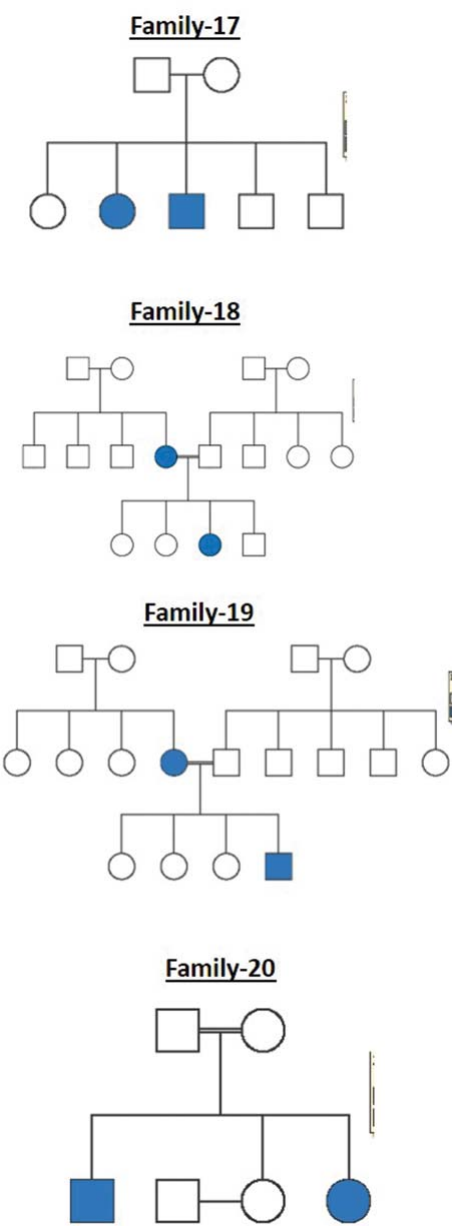
Pedigree chart for families 17, 18, 19, and 20.

Overall, 4 to 5 mL of venous blood was taken in the Ethylene diamine tetraacetic acid (EDTA) tubes. DNA was extracted from the blood lymphocytes using the instead of salting out. 14 The DNA isolation was done at Vasavi Medical Research Centre, Hyderabad. To assess the purity and concentration of extracted DNA, an ultraviolet (UV) spectrometer was used to calculate the average 260/280 nm. The ratio of absorbance readings at the two wavelengths should be between 1.8 and 2.0 (i.e., A260/A280 = 1.7–2.0). Later, the DNA was sent for SNP genotyping of the polymorphisms. The characteristics of the selected polymorphisms are shown in Table 1 .

**Table 1:** Characteristics of the SNPs

SNP	Gene	Functional consequence	Normal sequence	Ancestral allele
rs17715416	*MYO5B*	Intronic variant	A/G-FWD	A
rs6507992	*SKA1*	Missense variant	A/G-FWD	A
rs17713847	*MYO5B*	Intronic variant	A/G-FWD	G
rs6507872	*CTIF*	UTR variant	C/T-REV	C
rs8091995	*CTIF*	UTR variant	G/T-FWD	G
rs78950893	*SMAD7*	Intronic variant	C/T-FWD	C
rs1450425	*LOXHD1*	Intronic variant	A/G-REV	A
rs183559995	*MYO5B*	Intronic variant	A/G-FWD	G

Abbreviations: FWD, forward; REV, reverse; rs, reference SNP; SNP, single nucleotide polymorphism; UTR, untranslated region.

Note: The source of information for the nucleotide variants is available at: https://www.ncbi.nlh.nih.gov/snp/ and http://asia.ensembl.org/Homo_sapiens/Info/Index .

**Fig. 7:**
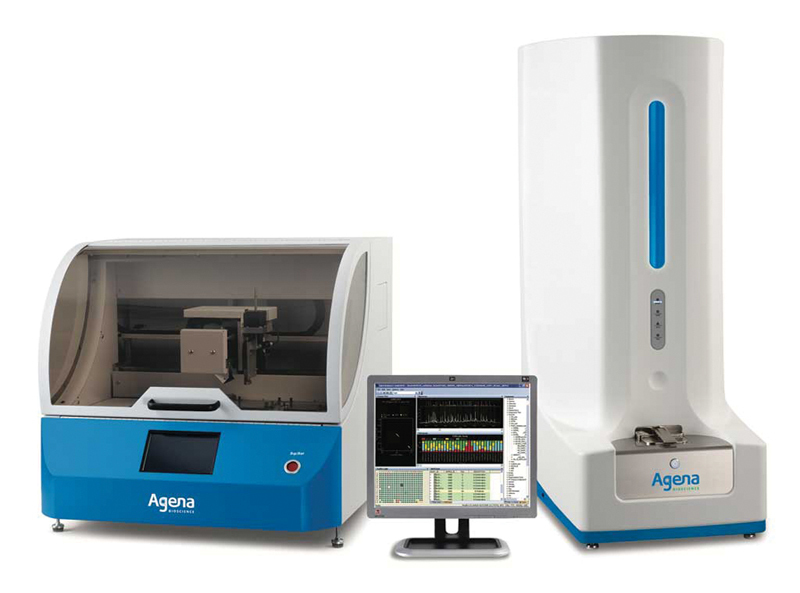
MassARRAY system.

Agena Bio MassARRAY (Agena Bioscience, Inc., San Diego, California, United States) platform using iPLEX Gold technology was utilized for the SNP genotyping. This system is highly accurate detection platform utilizing the Matrix-Assisted Laser Desorption/Ionization—Time of Flight (MALDI-TOF) mass spectrometry. Using proprietary Agena software (Assay Design Suite 2.0), the assay was designed for primers. Table 2 shows the primers and reverse sequence for all the SNPs along with the assay numbers. Following the correct workflow, according to the MassARRAY protocol, the samples are run through the analyzer. Agena's SpectroTyper 4.0 software (San Diego; California, United States) was used which automatically generates reports that identify the SNP alleles (homozygous or heterozygous). Fig. 7 shows the MassARRAY system. The data obtained from the analyzer software is sent for statistical analysis. MassARRAY analysis was done at Genes2Me, a subsidiary of Imperial Life Sciences, Delhi.

**Table 2:** Primers and reverse primer sequence for all the SNPs

Assay number	SNP	Primer sequence	Reverse primer sequence
Assay 1	rs728683	1-CCAGCCTACCTCATTTGTTG	2-CAGTGTGCAAATAGGGTAAG
Assay 2	rs17715416	1-CTTGTATCACATCCCTACCC	2-CTTGGACCTGTTGGATGAAG
Assay 3	rs6507992	1-GGAACTCTGTGAATCTCTTG	2-GGGTTACTGTTACTTGAGGC
Assay 4	rs17713847	1-ATGGGCAGCCGTCAGAAATG	2-ATCTCCCACTTCCTGAGTTC
Assay 4	rs6507872	1-CAGGTTGCTGCTCCTTTTTC	2-CGGGGTCTCAAATTTTAGGC
Assay 5	rs8091995	2-GGCCGAGTCGGTATTTATTC	1-TGATCTGTGGTACCCTTCCC
Assay 6	rs78950893	1-TAAAGGACTGCAGGATGAGG	2-CTGGTCTGGACTTCATTCTC
Assay 7	rs183559995	1-TTCTGGAGGTCATGGCTAAG	2-CATGGTGTCTGAAATGAGGG
Assay 8	rs1450425	1-AAGGGCAGAGGCCCAAATGA	2-GTGCCAGCTCTTCTTGGTTC

Abbreviations: rs, reference SNP; SNP, single nucleotide polymorphism.

### Statistical Analysis

The SNP allele data of the affected and controls obtained from the MassARRAY system was subjected to statistical analysis. PLINK software (version 1.09) was used for this study. 15 It is a free, open-source whole genome association toolset, designed to perform a range of basic to large-scale analyses in a computationally efficient manner. Genotype distribution was used to calculate the Hardy–Weinberg equilibrium (HWE) using the same PLINK. Statistical comparisons between the affected and unaffected were performed using PLINK software. Odds ratio (OR) and 95% confidence intervals were provided. Allelic association was analyzed using the Chi-square test. For nominal association, the statistical significance level is set to *α* = 0.05.

## Results

Eight high-risk variants (SNPs), that is, rs6507872 and rs8091995 of *CTIF* , rs17715416, rs17713847 and rs183559995 of *MYO5B* , rs78950893 of *SMAD7* , rs1450425 of *LOXHD1* , and rs6507992 of *SKA1* gene, present on 18q21.1 locus genotyped in all the multiplex families. All the polymorphisms follow HWE. In the allele association analysis ( Table 3 ), the *p* -value was >0.05. Hence, there was no significant difference in the allelic frequencies between NSCLP patients and healthy controls. The OR also was <1.6 in all the polymorphisms.

**Table 3:** Allelic association for SNPs

CHR	SNP	A1	F_A	F_U	A2	CHISQ	*p*	OR (95% CI)
18	rs1450425	T	0.34	0.3816	C	0.3248	0.5687	0.8349
18	rs17713847	A	0.05	0.06579	G	0.2009	0.654	0.7474
18	rs17715416	A	0.47	0.4211	G	0.4182	0.5178	1.219
18	rs6507872	T	0.09	0.07895	C	0.06766	0.7948	1.154
18	rs6507992	G	0.15	0.1184	A	0.3657	0.5454	1.314
18	rs728683	A	0.24	0.3158	G	1.251	0.2635	0.6842
18	rs78950893	T	0.04	0.02632	C	0.2456	0.6202	1.542
18	rs8091995	T	0.07	0.07895	G	0.05053	0.8221	0.8781

Abbreviations: A1, major allele (wild allele); A2, minor allele (mutant); CHISQ, Chi square; CHR, chromosome number; CI, confidence interval; F_A, minor allele frequency affected; F_U minor allele frequency unaffected; OR, odds ratio; rs, reference SNP; SNP, single nucleotide polymorphism.

Note: The *p* -values of <0.05 are significant.

## Discussion

Etiology of cleft lip and palate is polygenic and multifactorial. The various etiological factors include heredity, consanguinity, fetal environment, demographic factors, certain drugs, smoking and alcohol consumption during pregnancy, vitamins, infections, and malnutrition. Our understanding of the etiology and pathogenesis of nonsyndromic variants remains relatively inadequate. It is a reflection of the complexity and diversity of the mechanisms that involved at the molecular level during embryogenesis, with both genetic and environmental factors playing an important and influential role. With technological advancements in the field of molecular biology, our envelope of research on CLP has grown. Identification of genetic polymorphisms in our population would be invaluable in understanding the developmental mechanisms involved in causing the disease. Data from animal models, in which clefts arise either spontaneously or as a result of mutagenesis experiment, combined with an analysis of how expression patterns correlate with gene function and examining the effects of gene-environment interactions have proven themselves as powerful tools for identifying candidate genes for complex traits, like nonsyndromic clefts. Importantly, they also contribute to our knowledge of normal craniofacial development and the molecular pathogenesis of CLP, taking into account that facial development in mice mirrors human craniofacial development. Several recent studies have also provided strong evidence that syndromic forms having Mendelian patterns of inheritance may provide insights into the genetic etiology of nonsyndromic types of clefting.

Advances in the genomic arena led to whole-genome studies, linkage studies, and targeted studies. These led to the identification of many candidate genes in the etiology of CLP. Associations between polymorphic markers in *RUNX2, BMP4, TGFB3 PAX7, NTN1, IRF6, PTHFR, GHR* , etc., and risk of clefts were identified in different populations. Genetic studies conducted on different ethnicities both on syndromic and nonsyndromic cases revealed significant linkages on multiple chromosomes. Studies were conducted on case-parent trios, isolated clefts, and familial cases. However, studies on case-parent trios and familial cases or multiplex families are very less in India. When we take the percentage of familial cases, it comes to a meagre 3.5% of the total cleft cases. 4 The study sample was taken from a high-volume cleft center in India as people come from different states are treated. The nonsyndromic and familial cases were identified after a thorough medical history and examination of the patients. All syndromic cases were excluded during the data collection stage.

Though there is a standard protocol in the repair of CLP, the ultimate objective should be its prevention. Contemporary research pursuing environmental and genetic causes is underway and is concentrating on (1) environmental causes and (2) research on candidate genes, and susceptibility regions are underway in many parts of the world. In an article published by Mossey and Little, 5 in the INDIANCRAN (Indian collaboration on craniofacial anomalies) initiative, one of the recommendations was to study genetic polymorphisms and their disease susceptibility and association in various genetically identifiable groups in India.

Following the recommendations of INDIACRAN, we selected a susceptibility region 18q21.1 which revealed significant evidence in CLP. However, no study was reported in this susceptible region for its involvement in India and on familial cases of NSCLP. The SNPs evaluated are from candidate genes *CTIF* , *MYO5B* , *SKA1* , *SMAD7* , and *LOXHD1* . These genes are critical in protein synthesis, cell signaling, initiation, and targeting proteins to the plasma membrane.

The genetic studies in the short term will help in the evaluation and improve noninvasive methods of screening for the disorder in at-risk family members. The medium term helps in developing and establishing a knowledge-based approach to the management and treatment of individuals experiencing with CLP.

In the present study, 20 multiplex families were selected. The affected and unaffected patients participated were 50 and 38, respectively. Eight SNPs in the locus were analyzed for their association to cleft lip and palate in multiplex families. MassARRAY was selected for genotyping as it is a high throughput method having the multiplex capability, flexibility, adaptability, and the high level of accuracy. All the nucleotide variants showed no significance. The OR also was not more than 1.6.

In a study conducted by Beiraghi et al, 11 the SNP rs728683 showed a significant linkage for CLP. Fine mapping of the locus 18q21.1 revealed rs183559995 of *MYO5B* showed highly significant association with an OR of 18.09. The other SNPs, such as rs6507872 and rs8091995 of *CTIF* , rs17715416 and rs17713847 of *MYO5B* , rs78950893 of *SMAD7* , rs1450425 of *LOXHD1* , and rs6507992 of *SKA1* candidate genes, also showed significant evidence because of mutations leading to CLP, though with a different OR.

However, in the present study, none of the high-risk markers showed any significance. In a study conducted on two families on Indian population revealed significant evidence at 13q33.1–34. 16 This indicates that the marker identified as a risk, in one particular population or one large multigenerational family, may not be a significant marker in the same population or different population or some other families of the same population. The literature shows that some high-risk markers associated with CLP in some population were not involved with CLP, and in fact, reported as protective or decreased. In a study of the population in southern China, BMP4 rs17563 was reported to be a risk factor for cleft lip only. 17 According to Rafighdoost et al, 18 in the population of southeastern Iran, the BMP4 rs17563 variant has a protective effect on the occurrence of NSCLP. In a separate case-control study by Savitha et al, 19 on NSCLP, one study reported an increased risk of NSCLP in Indian population.

The variation or inconsistent results could be due to multifactorial etiology, epigenetic causes, and gene-gene interactions. The implications of the study suggest the multifactorial nature of the disease. The future research should be on the role of epigenetics, gene-gene interaction, and functional aspects of the polymorphisms.

## Conclusion

Within the limitations of this study, the high-risk SNPs rs6507872 and rs8091995 of *CTIF* , rs17715416, rs17713847 and rs183559995 of *MYO5B* , rs78950893 of *SMAD7* , rs1450425 of *LOXHD1* , and rs6507992 of *SKA1* genes present on locus 18q21.1 are not associated with the etiology of NSCLP in Indian multiplex families.
